# Forecasting Suitable Habitats of the Clouded Leopard (*Neofelis nebulosa*) in Asia: Insights into the Present and Future Climate Projections Within and Beyond Extant Boundaries

**DOI:** 10.3390/biology13110902

**Published:** 2024-11-05

**Authors:** Imon Abedin, Hilloljyoti Singha, Hye-Eun Kang, Hyun-Woo Kim, Shantanu Kundu

**Affiliations:** 1 Department of Zoology, Bodoland University, Kokrajhar 783370, India; 2 Institute of Marine Life Science, Pukyong National University, Busan 48513, Republic of Korea; 3 Department of Marine Biology, Pukyong National University, Busan 48513, Republic of Korea; 4 Marine Integrated Biomedical Technology Center, National Key Research Institutes in Universities, Pukyong National University, Busan 48513, Republic of Korea; 5 Department of Biology, Faculty of Science and Technology, Airlangga University, Surabaya 60115, Indonesia; 6 Ocean and Fisheries Development International Cooperation Institute, College of Fisheries Science, Pukyong National University, Busan 48513, Republic of Korea; 7 International Graduate Program of Fisheries Science, Pukyong National University, Busan 48513, Republic of Korea

**Keywords:** carnivore, feline, predator, species distribution model, corridor, conservation

## Abstract

Terrestrial carnivores, such as the clouded leopard (*Neofelis nebulosa*), are ‘vulnerable’ and experiencing significant population declines in mainland Asia. Considering the assessed threats, the IUCN has repetitively revised the range of this felid and now characterized it into four distinct categories (extant, possibly extant, possibly extinct, and extinct). Although several ecological works have been accomplished on this enigmatic carnivore, the overall view of its habitat suitability, fragmentation, and corridor connectivity in both present and historical ranges is lacking. Thus, achieving this ecological information under present and future climate scenarios is crucial. The model identified merely 44,033 sq. km falling within the extant range (representing 31.66%) and 20,034 sq. km (8.13%) in the possibly extant range. Fascinatingly, within the historical ranges, an additional 15,264 sq. km (6.58%) has been identified as suitable habitat in the possibly extinct range and 14,022 sq. km (2.38%) in the extinct range. Notably, a total of 25,614 sq. km of suitable habitat is found within designated protected areas across the entire range. Nevertheless, climate change is expected to drive habitat loss of up to 41% (overall IUCN range) for *N. nebulosa* in both present and historical extent, with habitat patches becoming increasingly fragmented. This is reflected in a projected decline in the number of viable habitat patches (NP) by up to 23.29% in the future. This study also identified 18 transboundary biological corridors for *N. nebulosa*, with Southeast Asian countries expected to experience the most significant declines in corridor connectivity. In contrast, the South Asian countries (Bhutan, Nepal, and India) are projected to maintain relatively higher connectivity in the future. Nevertheless, a substantial decline in overall mean corridor connectivity is projected in the near future due to the impacts of climate change. This study underscores the urgent need for a coordinated and multifaceted conservation strategy for *N. nebulosa*, focusing on mitigating habitat loss and fragmentation. Practical measures must be implemented to protect the species’ shrinking range, considering its declining corridor networks and heightened vulnerability to inbreeding depression. Moreover, the assessment of habitat suitability both within and beyond the extant range, alongside corridor connectivity measures, provides valuable insights into potential translocation and reintroduction sites for this species. These findings provide a critical foundation for developing a strategic conservation plan tailored to the specific needs of this felid species across South and Southeast Asia, ensuring enhanced climate resilience and mitigating associated threats.

## Simple Summary

The clouded leopard (*Neofelis nebulosa*) is listed as “vulnerable” due to significant population declines across mainland Asia. Despite existing ecological research, the understanding of the species’ habitat suitability, fragmentation, and corridor connectivity remains limited. The present investigation recognizes that, in addition to habitat suitability within its current extent, the species’ historical ranges also encompass a significant proportion of suitable habitats. Climate change is expected to exacerbate habitat loss, intensifying fragmentation and reducing the number of viable habitat patches. This study highlights the importance of several transboundary biological corridors, with Southeast Asia predicted to experience the most pronounced connectivity declines, while Bhutan, Nepal, and India are projected to retain more robust ecological networks. To address these challenges, a coordinated conservation strategy is crucial, focusing on mitigating habitat loss and fragmentation as well as addressing the species’ shrinking range and increased vulnerability to inbreeding. This study provides critical insights into potential translocation and reintroduction sites, laying the groundwork for a targeted conservation plan designed to protect the clouded leopard across South and Southeast Asia in response to climate change.

## 1. Introduction

Biodiversity is a critical component of Earth’s ecosystems, contributing a wide array of resources and playing an essential role in maintaining ecosystem stability and functionality [1]. However, it is increasingly under threat from a variety of anthropogenic drivers, such as deforestation, expansion of agriculture, urbanization, overexploitation of resources, etc. [2]. Besides these, climate change has also become a major factor exacerbating biodiversity loss, intensifying the impacts of ongoing human-driven land use changes and their associated risks [3]. The alterations in climate have led to significant reductions in habitat availability and increased landscape fragmentation, both of which negatively affect biodiversity at multiple scales [4]. These processes have contributed to a large-scale decline in species richness and ecosystem complexity, with numerous species facing habitat loss and intensified extinction risks, particularly those with small or fragmented populations [5,6].

As environmental conditions shift, many species have begun to migrate in an effort to track suitable ecological niches, a trend that is projected to accelerate in response to continued climate change [7,8]. Furthermore, the species that are unable to relocate or adapt to these rapid environmental changes are at an increased risk of local or global extinction [9]. As a result, the fragmentation of habitats is further disrupting ecological connectivity and increasing population isolation [4]. This connectivity is essential for enhancing the long-term resilience of species, as it facilitates gene flow and demographic stability, both of which are critical for maintaining population viability over extended periods [10,11]. Therefore, ensuring ecological connectivity is critical not only for the local conservation of species but also for supporting range shifts and adaptations in response to future threats such as land use changes and further climate disruptions [12,13].

This scenario of global biodiversity loss is particularly alarming for terrestrial carnivores, which have experienced significant population declines [14,15]. These species are especially sensitive to habitat fragmentation due to their large home ranges and typically low population densities [16,17,18]. Moreover, their vulnerability is further amplified by small population sizes, slow reproductive rates, extensive space requirements, dependence on stable prey populations, and frequent conflicts with humans [19,20]. Furthermore, this is especially concerning as terrestrial carnivores play vital roles in maintaining ecosystem balance, promoting overall ecosystem health, and regulating populations at lower trophic levels [21,22]. Despite their ecological importance and central role in food webs, terrestrial carnivores have experienced significant declines in both population size and geographic range over the past century, often leading to cascading effects such as decreased biodiversity and reduced species richness within ecosystems [23,24]. Given the mounting pressures from anthropogenic disturbances, habitat loss, fragmentation, low reproductive rates, and climate change, these species are ideal focal points for conservation efforts, as their populations have plummeted significantly [25,26,27,28].

The clouded leopard (*Neofelis nebulosa*) is a medium-sized felid and remains the least studied among the larger wildcats, largely due to its elusive nature, nocturnal habits, and low population density [29]. Morphologically, this species has a dark gray or ochreous coat with distinctive large, irregular dark blotches, forming a cloud-like pattern [30]. The clouded leopard exhibits a strong arboreal tendency and is predominantly associated with forest habitats, particularly primary evergreen tropical rainforests, while also being recorded in deciduous and mixed forest ecosystems [31]. Additionally, the species has been recorded at elevations exceeding 3500 m in the Himalayas [32]. Furthermore, this species serves as an umbrella species for the Asian forest ecosystem, with a distribution that extends from the foothills of the Himalayas in Nepal, Bhutan, and India, across South China, and down to Peninsular Malaysia [33,34]. However, *N. nebulosa* is now extinct on the island of Taiwan, and populations in Vietnam, China, and Bangladesh are critically low, with some areas facing the risk of extirpation [31,35]. Furthermore, in its 2021 assessment, the IUCN Red List classified *N. nebulosa* as ‘vulnerable,’ a status that reflects a significant decline in populations across much of its range. This decline is attributed to multiple factors, including habitat destruction, direct exploitation, targeted hunting, and incidental mortality from snares intended for other wildlife [31]. The clouded leopard is listed under CITES Appendix I and is protected by national wildlife laws across its range in various countries. Despite legal protections, body parts of the *N. nebulosa* continue to appear in illegal trade, with evidence indicating that both targeted poaching and incidental capture in snares contribute to population declines. Moreover, it is estimated that populations of *N. nebulosa* have decreased by over 30% between 1999 and 2019, particularly in Myanmar, Cambodia, Laos, Vietnam, Bangladesh, and China. However, these countries likely harbored substantial populations in previous decades, but recent biodiversity loss in the region has significantly impacted these species and adversely affected their numbers. Furthermore, population trends in other range countries, such as Nepal, Bhutan, India, Malaysia, and Thailand, remain inadequately documented, underscoring the need for further research and targeted conservation strategies to ensure the survival of this elusive felid [31].

Prior to this study, most ecological research on *N. nebulosa* centered on its occupancy, habitat use, range, and behavior within fragmented areas of South and Southeast Asia [27,31,36,37,38]. However, key issues such as the potential impacts of climate change, habitat fragmentation, and the importance of corridor connectivity have yet to be thoroughly explored for this elusive carnivore. Moreover, the diet of this species includes a broad spectrum of prey, such as primates, small mammals, ungulates, and Galliformes. However, studies have highlighted concerns that the distribution of these sympatric prey species may shift due to habitat loss driven by climate change, which could significantly impact the *N. nebulosa* [39,40,41]. Thus, understanding habitat suitability and connectivity within the range of *N. nebulosa* is essential for effective management, particularly given the increasing anthropogenic pressures on natural habitats and the looming threats posed by climate change [42,43,44,45]. Consequently, many studies have utilized species distribution models (SDMs) to assess habitat suitability and connectivity for various Felidae species [46,47,48]. These models quantify the relationships between species and their environments to determine habitat suitability and can aid in monitoring population dynamics, community ecology, and spatial management planning [49,50,51]. Furthermore, ensemble modeling has emerged as a more robust and reliable method for predicting species distribution compared to individual SDM approaches [52,53,54]. This methodology enhances predictive accuracy by integrating outputs from multiple models, thereby reducing reliance on any single model that may not be optimal for addressing specific research objectives [55]. These approaches yield essential insights into the species’ habitat within and beyond the IUCN-defined extant range, thereby enhancing the conservation strategy not only for its current distribution but also for potential habitats outside this range. Consequently, the current study utilizes SDM within the categorical IUCN range of *N. nebulosa*, focusing on both the existing (extant and possibly extant) and the likely historical ranges (possibly extinct and extinct) in present and future climatic scenarios to (a) identify suitable habitat extent and its dynamics; (b) assess the fragmentation of suitable habitats; and (c) evaluate corridor connectivity within and beyond its present extent. These approaches will facilitate the conservation of this species within its existing ranges and support the assessment and development of management plans for habitats resembling historical ranges, thereby enhancing the protection of the species in the wild.

## 2. Materials and Methods

### 2.1. Study Area and Species Occurrence Records

**Figure 1:**
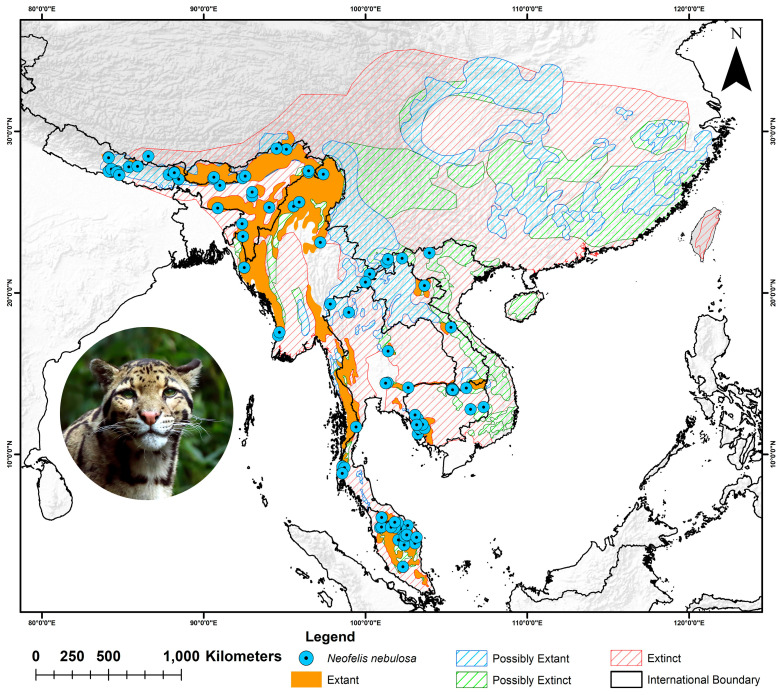
The figure illustrates the entire IUCN extant range of the clouded leopard (*Neofelis nebulosa*), divided into four distinct ranges across South and Southeast Asia, with presence points gathered through primary field surveys and secondary sources. The photograph of the clouded leopard was retrieved from the web.

The clouded leopard is distributed from the Himalayan foothills in Nepal through mainland Southeast Asia into China [56] (Figure 1). However, the species is extinct on the island of Taiwan, and populations are critically low, with near-extirpation in Vietnam, China, and Bangladesh [35]. As a result, the IUCN range is divided into four categories: extant, possibly extant, extinct, and possibly extinct [31]. The extant range covers much of Bhutan, Northeast India, Myanmar, Thailand, Cambodia, Laos, and the Malay Peninsula, with small fragments in Nepal and southern Bangladesh. In contrast, China, despite having the largest area within the species’ range, falls under the historical ranges and is not part of the extant range (Figure 1). Similarly, Vietnam is classified under both the extinct and possibly extinct ranges for this species. The criteria for dividing the species’ entire range into four distinct categories were based on the population decline rate and detection frequency in each region. For example, in countries like Vietnam, Laos, and China, populations had already decreased by over 60% by 1999, and recent assessments of 2021 assumed that these areas may no longer support any viable populations. Additionally, some regions reported extremely low population numbers and detection rates, which is considered a ‘possibly extinct’ category. However, some regions lacked population estimates, and the species has not been reported for a long time, classifying these areas as ‘extinct’ range. Interestingly, in certain regions, sporadic sightings from camera traps or studies on other large carnivores suggest that the species may still persist there, warranting their classification as ‘possibly extant’ range. Additionally, areas with recent and regular sightings were categorized as ‘extant’. This study utilized a dataset consisting of 159 location points, of which nine were obtained from primary field visits, while 151 were sourced from secondary data via the GeoCat website (accessed on 16 August 2024), aggregating information from GBIF and iNaturalist [57]. Additionally, in order to ensure the accuracy and ecological relevance of the dataset, records related to preserved specimens and individuals held in captivity were deliberately excluded from the analysis. The location points from primary studies were collected during opportunistic visits to forests of northeast India, specifically from the Mathanguri area of Manas National Park (*n* = 2), Jeypore rainforest (*n* = 3), and Soraipung (*n* = 2) in Dihing Patkai National Park in Assam state. Additionally, sightings were recorded in Namdapha National Park near the Deban area (*n* = 2) in Arunachal Pradesh. The spatial correlation was evaluated and constrained through the application of the spatial rarefaction function in SDM Toolbox v2.4, utilizing a resolution of 4.5 km to correspond with the raster data pixel size [58]. This approach reduced the potential for model overfitting and enhanced the robustness and accuracy of the analysis [59]. As a result, following the removal of spatially correlated occurrences, 148 unique location points were included in the analysis. Furthermore, to evaluate the extent of protected areas within this landscape, data were obtained from the Protected Planet website (https://www.protectedplanet.net/en) (accessed on 22 August 2024) [60].

### 2.2. Distribution Predictors for the Clouded Leopard

This study employed a comprehensive set of covariates to model habitat suitability for *N. nebulosa*, incorporating bioclimatic, topographic, habitat, and anthropogenic factors. A total of 19 bioclimatic variables, including temperature, precipitation, and other climate-related factors that influence species distribution, were sourced from WorldClim (https://www.worldclim.org/) (accessed on 22 August 2024) [61]. The topographic variables, such as elevation and slope, were obtained from NASA’s Shuttle Radar Topographic Mission (SRTM) with a spatial resolution of 90 m (http://srtm.csi.cgiar.org/srtmdata/) (accessed on 22 August 2024). Furthermore, the habitat variables were selected based on the IUCN Red List, focusing on evergreen and mixed-deciduous forests, which represent key environments that meet the ecological needs of the species. Additionally, to assess the influence of human activity, anthropogenic variables such as built-up/urban areas and cropland were included. The habitat variables were derived from the Copernicus Global Land Service land use and land cover (LULC) categorical raster and transformed into continuous raster datasets using Euclidean distance in ArcGIS, enabling detailed analysis of species’ responses to habitat proximity [62,63]. Furthermore, all variables were resampled to a uniform resolution of 2.5 min (approximately 4.5 km^2^) using ArcGIS 10.6 to ensure consistency across datasets and improve the accuracy of the analysis. Moreover, to address potential multicollinearity, the SAHM (Software for Assisted Habitat Modeling) package in VisTrails was used [64]. The variables with a correlation coefficient (r) greater than 0.8 were excluded to avoid redundancy [65] (Figure S1). After this process, 12 variables were retained for the final model, ensuring robust and accurate predictions of habitat suitability.

### 2.3. Assessment of Future Climate Change Projections

In order to evaluate the potential impacts of climate change under two distinct shared socioeconomic pathways (SSPs)—SSP245 and SSP585—this study utilized future climate projections for the periods 2041–2060 and 2061–2080. These SSPs serve as frameworks for examining various trajectories of socioeconomic development and their corresponding effects on greenhouse gas emissions and climate change dynamics. The SSP245 scenario projects a future characterized by moderate mitigation efforts, including balanced environmental and social policies, moderate population growth, and advancements in technology, whereas the SSP585 scenario delineates a high-emissions trajectory characterized by minimal adaptation measures, rapid population growth, elevated energy demands, and insufficient environmental regulation, resulting in continued emissions throughout the century [66,67]. The Hadley Centre Global Environment Model, Global Coupled Configuration 3.1 (HadGEM3-GC31 LL), part of the sixth Coupled Model Intercomparison Project (CMIP6), was chosen for its strong performance in modeling climate variability and temperature distribution in South and Southeast Asia [68,69]. To isolate the effects of climate change in future projections, non-climatic variables—including habitat types such as evergreen and mixed forests, urban areas, and cropland, as well as elevation and slope—were held constant that restricted species distribution probabilities to ecologically relevant areas for this species [70,71]. A comprehensive national-scale assessment of habitat suitability was conducted, which integrated the legal frameworks of each country to inform targeted conservation strategies, assessed utilizing the zonal statistics function in ArcGIS v.10.6 to systematically evaluate suitable habitats within the species’ range across multiple countries.

### 2.4. Species Distribution Model for Clouded Leopard

The habitat suitability modeling in this study employed an ensemble approach, integrating multiple algorithms to create the final model for the species utilizing five distinct algorithms: Boosted Regression Tree (BRT), Generalized Linear Model (GLM), Multivariate Adaptive Regression Splines (MARS), Maximum Entropy (MaxEnt), and Random Forest (RF) [72,73,74]. The selection of these models captures various aspects of species–environment relationships. Specifically, MaxEnt handles presence-only data, BRT and RF manage non-linear interactions with high accuracy, while GLM and MARS offer interpretability and flexibility, creating a comprehensive modeling approach. These algorithms were implemented through the SAHM package in VisTrails software, resulting in probability maps that ranged from 0 (least suitable) to 1 (most suitable), and binary maps were subsequently generated using the minimum training presence as a threshold [64,75]. The evaluation of the models was based on an Area Under the Curve (AUC) threshold of 0.75, which served as the primary criterion for model selection [76]. Moreover, to evaluate habitat configurations, an ensemble count map was generated with a scale from 0 to 5, where each pixel indicated the level of agreement among the models, with a value of 5 signifying complete accord across all models. Additionally, several performance metrics were calculated for both the training and cross-validation datasets (*n* = 10) to further evaluate model performance. Furthermore, metrics such as AUC, True Skill Statistic (TSS), Cohen’s Kappa, Proportion Correctly Classified (PCC), specificity, and sensitivity further ensured a robust and reliable model for predicting the species’ distribution [77,78,79,80].

### 2.5. Assessment of Habitat Quality and Shape Geometry

To assess the qualitative and geometric characteristics of suitable habitat patches in both current and projected future scenarios, this study employed a range of class-level metrics using FRAGSTATS version 4.2.1 [81]. This specialized software is well-established in the fields of landscape ecology and environmental management, facilitating the analysis of spatial patterns within ecosystems and offering an extensive array of metrics and indices that thoroughly assess and clarify the structure and composition of landscapes [82]. The metrics utilized in this analysis included the number of patches (NP), aggregate index (AI), patch density (PD), largest patch index (LPI), total edge (TE), and landscape shape index (LSI). The metrics such as NP, PD, TE, and LPI offer detailed insights into the geometry of habitat patches, including their size, edge characteristics, and density within a specific area. In contrast, the LSI metric assesses the complexity of patch geometries, indicating the level of irregularity or convolution present in their shapes, whereas the AI quantifies the closeness or clustering of patches, reflecting the extent to which they are grouped or spread out across the landscape. These metrics hold biological significance, as they provide insights into ecological processes within habitats and elucidate the effects of changes in suitable areas on landscape dynamics [83]. This methodology allows for a deeper understanding of landscape characteristics and facilitates a comprehensive analysis across the species’ distribution range. Consequently, these metrics were applied to evaluate habitat features and levels of fragmentation within the modeled area under various scenarios, including current conditions and future climate change projections [62].

### 2.6. Assessment of Biological Corridor Connectivity

Recognizing the importance of enhancing habitat connectivity as a vital conservation strategy for species preservation, it was essential to evaluate the biological connectivity between habitat patches [84]. Hence, to accomplish this, the circuit model—frequently used for designing animal corridors—was employed. Specifically, the Circuitscape toolbox for ArcGIS 10.6 was utilized to simulate these corridors in present and future climatic scenarios [85,86,87]. This toolbox allows for the simulation of ecological corridors by modeling species movement across heterogeneous landscapes. In this framework, pairwise source/ground mode settings were applied using the probability maps generated by the model as conductance rasters. The specified location points served as focal nodes within the pairwise configuration. The output comprised current maps, which facilitated a more in-depth assessment and analysis of connectivity. This simulation of corridors was executed for both contemporary conditions and projected future climatic scenarios.

## 3. Results

### 3.1. Ecological Niche Modelling and Predictor Importance

**Figure 2:**
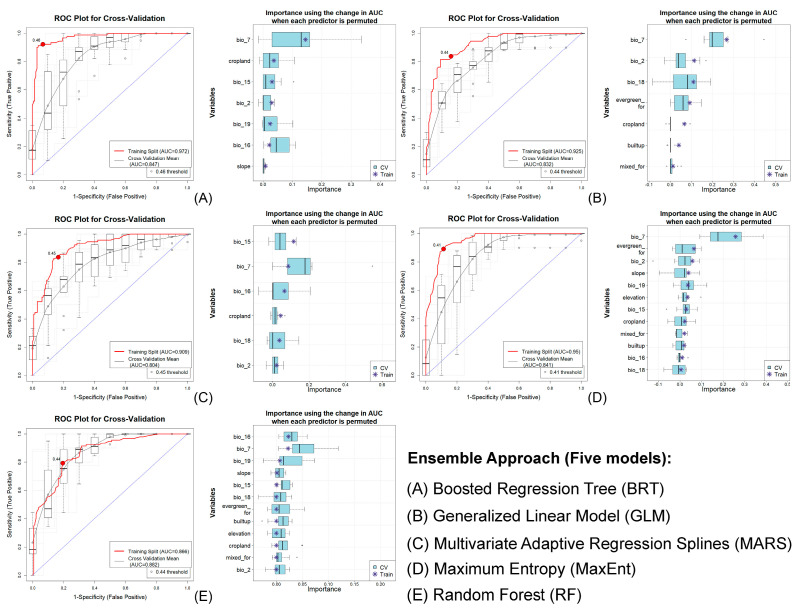
Model evaluation plot showing the average training ROC of both training and cross-validation (CV) and the predictors chosen by the model for the replicate runs under five models.

The models’ performance for this species was consistently outstanding across all algorithms, as evidenced by results from both training and cross-validation datasets. Among the five models employed, MaxEnt utilized the complete set of variables during replication, whereas the BRT model incorporated only six out of the twelve available variables, highlighting their contrasting abilities to assimilate data for habitat prediction. Moreover, all algorithms with an AUC score above 0.75 were included in the final ensemble map, with AUC values ranging from 0.972 to 0.804 during training and 0.909 to 0.804 during cross-validation, indicating high predictive accuracy (Figure 2, Figures S2 and S3). The Generalized Linear Model (GLM) had the smallest ΔAUC (0.093), while the Boosted Regression Tree (BRT) showed the largest deviation (ΔAUC up to 0.125), highlighting model sensitivity. Additionally, evaluation metrics such as True Skill Statistic (TSS), Proportion Correctly Classified (PCC), Cohen’s Kappa, sensitivity, and specificity further validated the models’ high performance (Table 1).

The ensemble model analysis indicated that on average (μ) across the five models, temperature annual range (bio_7) was the most influential predictor of species distribution, contributing 37.37% to overall model performance (Table 2). This variable, representing the difference between the highest temperature in the warmest month and the lowest in the coldest, serves as a key measure of temperature variability and seasonal extremes affecting species distribution and habitat suitability. Among habitat variables, Evergreen Forest had the highest influence, contributing 9.74%, while the anthropogenic variable Cropland contributed 8.19% to the model. Among the topographic variables, slope had the highest contribution, accounting for 2.35%. Conversely, the habitat variable Mixed/Deciduous Forest (mixed_for) had the lowest contribution, at 2.10% (Figure 2 and Figure S4, Table 2).

### 3.2. Habitat Suitability in Present and Historical Range

**Figure 3:**
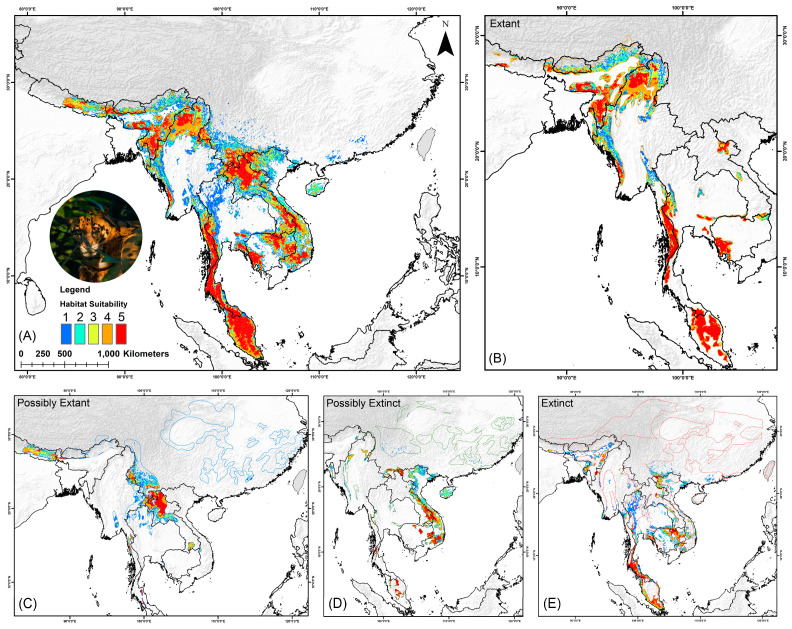
This figure displays the present suitable habitats for the clouded leopard in the study area. The five classes (1–5) on the map represent the model agreements used in the study, while Class “0” (no suitability and zero model agreement) is not shown. The map sections are as follows: (**A**) Habitat suitability across the entire IUCN range, (**B**) extant range, (**C**) possible extant range, (**D**) possibly extinct range, and (**E**) extinct range. The photograph of the clouded leopard was taken by Dhritiman Mukherjee.

The total IUCN range for the species, encompassing all four categories, spans approximately 1,201,540 sq. km across South and Southeast Asia, of which only 93,353 sq. km, or 7.76%, was identified as suitable habitat in the current scenario (Figure 3, Table 3). Furthermore, the model identified 44,033 sq. km suitable within the extant range, representing 31.66% of the area, whereas in the possibly extant range, only 20,034 sq. km, or 8.13%, was found to be suitable. Additionally, in the extinct range, only 14,022 sq. km, or 2.38%, still exhibited suitability, while in the possibly extinct range, 15,264 sq. km, or 6.58%, was identified as suitable habitat. Nevertheless, only 25,614 sq. km of suitable habitat within the entire range was found to be under protected areas (Table 3).

**Figure 4:**
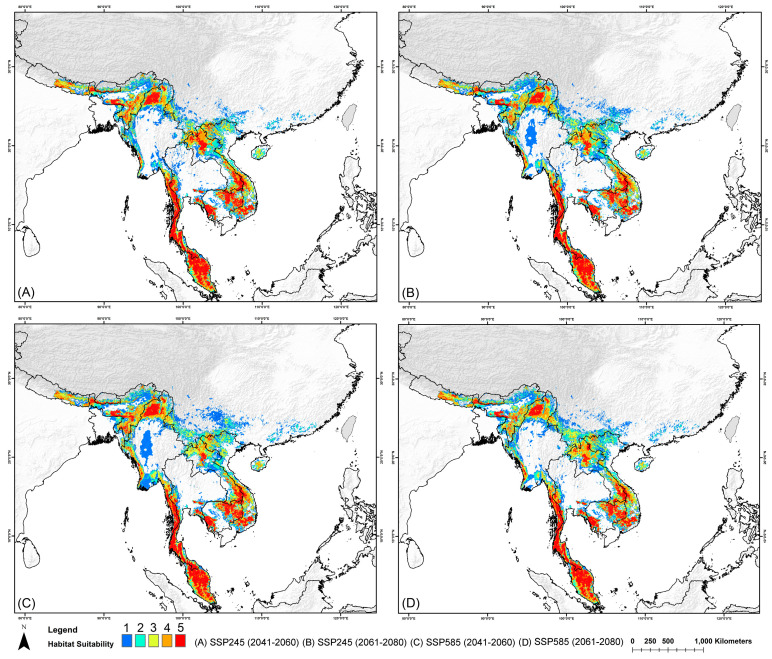
Map illustrating habitat suitability for the clouded leopard across the entire IUCN range under future climate change scenarios. The subfigures represent different scenarios and timeframes.

In future scenarios, the suitable habitat is projected to decline across all ranges due to climatic changes. Overall, the entire IUCN range is expected to experience a decline of over 26.97% from present conditions (Figure 4). Specifically, in the extant range, a reduction of 14% to 27% is expected, while the possibly extant range is projected to experience a substantial decline of up to 80.83%. Similarly, the extinct range is forecast to undergo a reduction of over 21%, and the possibly extinct range is expected to decrease by up to 37.34% (Figure 4). Moreover, suitable areas within PAs are projected to decrease by approximately 17.17% due to climate change impacts (Table 3).

### 3.3. Country-Level Mean Habitat Suitability

The analysis of mean habitat suitability across the species’ range nations indicates that Malaysia currently exhibits the highest mean suitability (0.743), followed by Laos (0.493), India (0.488), and Cambodia (0.410) (Table 4). In contrast, China shows the lowest suitability (0.098), with no suitable habitat identified in Taiwan. As climatic changes are projected to reduce suitable habitat extent, these shifts are expected to have a substantial impact on the overall quality of habitat throughout the species’ range. The most significant decline is forecasted for India, where mean suitability may decrease by as much as 56.12% from present levels, followed by Myanmar, where reductions of 32% to 41% are anticipated (Table 4). Moreover, the mean habitat suitability across all nations within the *N. nebulosa* range is anticipated to decline in the future due to climate change. However, among the nations currently deemed suitable, Malaysia stands out with the smallest expected decrease, facing a reduction of only 12.59% in the future. In comparison, China is predicted to experience the least overall decline, with suitability dropping by 5% to 15% across different future scenarios.

### 3.4. Habitat Quality and Shape Geometry

The analysis of habitat shape geometry indicates significant fragmentation driven by future climate changes. A high degree of disintegration is observed as habitat patches become fragmented, with some viable patches completely lost (Table 5). This is reflected in a decline in the number of patches (NP) by up to 23.29% in the future from the present scenario. Furthermore, the reduction in patch density (PD), which has decreased by over 8.10%, underscores the extent of this fragmentation. In addition to the loss of patches, the remaining patches are now smaller in size, as evidenced by a substantial decline of 24.50% in the LPI. Consequently, the edge area is also diminished, with total edge (TE) declining by more than 18.01%. Moreover, the remaining patches exhibit simpler shapes, as indicated by a decrease of up to 13.22% in the LSI (Table 5). The spatial arrangement of these patches has also changed, with increased distances between them, as demonstrated by a decline of over 14% in the AI. Collectively, these metrics comprehensively illustrate and elucidate the fragmentation of suitable habitat patches induced by climate change.

### 3.5. Biological Corridor Connectivity

**Figure 5:**
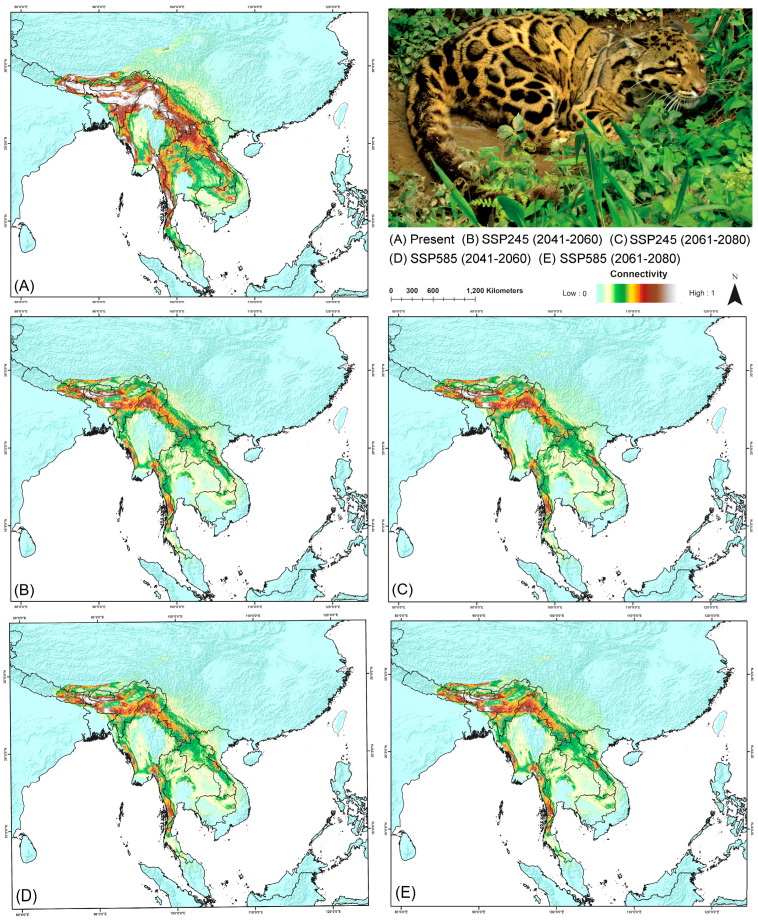
Map depicting (**A**–**E**) the connectivity of the clouded leopard across its entire IUCN range in Southeast Asia under current and future climatic scenarios. The photograph of the clouded leopard was taken by the first author (I.A.).

The assessment of biological corridor connectivity revealed 18 transboundary corridors for this species in its IUCN range (Figure 5). The highest mean corridor connectivity was observed between Bhutan and India, with a connectivity score of 2.441 in the current scenario (Table 6). This is followed by the Nepal–India corridor (2.114), Myanmar–China (1.140), and Nepal–China (1.044). The lowest connectivity was recorded between Thailand and Malaysia, with a mean score of 0.253 (Table 6). However, these transboundary corridors are projected to be significantly affected by climate change in the future (Figure 5). The most substantial decline in mean corridor connectivity is anticipated between Bhutan and China, with reductions of up to 61% in the future from the present scenario. However, other high-connectivity corridors are also expected to experience decreases, with the Bhutan–India corridor projected to decline by 15% to 31% and the Nepal–India corridor by up to 29.53% from the present scenario. The highest decline in the highest mean corridor connectivity is demonstrated by the Southeast Asian countries, while South Asian nations such as Bhutan, Nepal, and India are expected to maintain relatively higher connectivity compared to their counterparts in the future (Figure 5). Nonetheless, an overall loss of mean corridor connectivity for this species is anticipated, primarily driven by the impacts of climate change.

## 4. Discussion

Given the ongoing global biodiversity loss, terrestrial predator populations are increasingly impacted by both environmental changes and direct and indirect anthropogenic pressures [15]. In this context, identifying the extent of suitable habitat for carnivore species—considering bioclimatic, ecological, and human-induced factors—becomes essential for species conservation [27]. This study represents the first attempt to assess the impact of climate change on the habitat and corridor connectivity of *N. nebulosa* across its present and historical range. The findings of this study will contribute to the development of more effective and comprehensive management plans for the species, addressing the threats it faces in the future.

The forests of South and Southeast Asia are undergoing significant deforestation, and this ongoing habitat loss, combined with climate change, poses a substantial threat to the survival of *N. nebulosa* [88]. The model identified a strong affinity of this species for evergreen forest, which contributed 9.74%, making it the highest contributing habitat variable. This result corroborates previous field studies indicating that *N. nebulosa* predominantly inhabits evergreen forests over other vegetation types [33,89]. In contrast, the contribution of mixed/deciduous forest habitats was relatively low, accounting for only 2.10%, highlighting the species’ specific habitat preferences. This finding underscores the species’ distinct habitat preferences and aligns with previous research, which identified it as primarily an evergreen forest dweller [31]. Moreover, the low contribution may reflect the fact that sightings in deciduous or mixed forests are very rare and occasional, which further reinforces the conclusion that the species is largely confined to evergreen forest ecosystems. Among the bioclimatic variables, “Temperature Annual Range” (bio_7) was identified as the most significant factor, accounting for 37.37% of the model’s contribution. This aligns with prior research highlighting the critical importance of this variable for medium-sized felids [47,48]. Additionally, previous studies have underscored the importance of primary forest habitats and topographic variables, such as slope, which also demonstrated a relatively high contribution in the assessed model. Additionally, elevation is another significant variable, as *N. nebulosa* is known to utilize higher altitudes to evade larger predators [90,91,92]. Hence, the present study meticulously revealed the importance of each variable in the distribution of this species in its range.

Among the four distinct categories in the IUCN designated ranges, the majority of this suitable habitat is concentrated within the extant (44,033 sq. km) and possibly extant ranges (20,034 sq. km). However, it is noteworthy that substantial portions of suitable habitat were also found within regions classified as possibly extinct (15,264 sq. km) and extinct (14,022 sq. km). This finding is particularly intriguing, as these areas may still hold ecological value and should be prioritized for field validation. Although these regions may not currently support any individuals of *N. nebulosa*, there remains the possibility that they offer the necessary habitat conditions. As a result, these areas could be considered potential sites for species reintroduction or relocation efforts, offering an opportunity to expand the species’ range and ensure its long-term persistence. Furthermore, a significant portion of the identified suitable habitat falls within PAs, particularly under the extant range, emphasizing the critical role these areas play as key refuges for the species. The presence of suitable habitats within PAs highlights their importance for ongoing conservation efforts, as they provide secure environments free from human disturbance. However, this study also reveals that climate-induced habitat loss poses a severe threat to the species across all its ranges. The entire species range is expected to experience a considerable decline in suitable habitat due to future climate scenarios. Despite this, the extant range is projected to undergo the least severe reduction, with a decline of up to 27.71%. This emphasizes the urgent need to focus conservation efforts on these crucial habitats, as they represent the last strongholds for the species. Therefore, any additional human-induced impacts, such as deforestation or habitat degradation, could further deteriorate the precarious status of species, making immediate protection and conservation of these areas’ paramount. These findings are consistent with previous research that has documented the significant decline in suitable habitats and species ranges due to the ongoing loss of tropical forests in the region [93,94,95]. The countries that currently exhibit relatively high levels of habitat suitability for the species, such as Malaysia, Laos, and India, should be prioritized for conservation, as they represent critical areas for the species’ survival in the future. However, these nations are also projected to experience substantial losses in habitat quality due to deforestation, land-use change, and climate change. The continued fragmentation of tropical forests in the region further complicates this scenario, as it isolates suitable habitats and increases the risk of population decline [96].

*N. nebulosa* is projected to face significant habitat fragmentation across its entire range, largely driven by climate-induced changes. This is evidenced by the anticipated decline in the number of viable habitat patches, as indicated by the reduction in NP in future scenarios. Furthermore, these patches are expected to decrease in size, as reflected by the declining LPI, and the density of occurrences will be much lower, as shown by the reduced PD. In addition to these changes, the patches will exhibit simpler shapes with less edge complexity, as indicated by decreases in TE and LSI. The fragmentation is further aggravated by the increased proximity of these patches to one another, as revealed by the decline in AI. Overall, the habitat patches are predicted to become smaller, more simplified, and more isolated, a concerning trend supported by previous field studies [97]. This increasing habitat fragmentation is likely to severely impact corridor connectivity for the species, limiting its ability to move between habitat patches and threatening its long-term survival within its range.

The present study identified 18 transboundary biological corridors for this species within its present and historical extent. Notably, the findings indicate that the highest mean corridor connectivity occurs between Bhutan and India, followed closely by the Nepal–India corridor. This elevated connectivity, particularly in the foothills of Bhutan, can be attributed to the presence of extensive, contiguous forested areas, many of which are protected under the administrations of both India and Bhutan. Specifically, areas such as Manas National Park, Royal Manas National Park, Phibsoo Wildlife Sanctuary, Raimona National Park, and Buxa Tiger Reserve, extending continuously toward Nepal, contribute significantly to this high connectivity, providing critical habitat for the species. In contrast, this study revealed that the lowest connectivity was found between Thailand and the Malay Peninsula. This may be largely due to physical barriers, such as the Isthmus of Kra, which interrupts habitat continuity in the region. Furthermore, it is important to highlight that these biological corridors are increasingly vulnerable to the impacts of climate change, habitat loss, and fragmentation. The loss of connectivity poses a substantial risk to this predator, which requires large, uninterrupted movement ranges for ecological activities [98]. Moreover, a significant portion of this decline in corridor connectivity is occurring in Southeast Asian countries, a region that is particularly susceptible to the combined effects of rapid climate shifts and escalating anthropogenic pressures [99,100]. Thus, this study underscores the urgent need for targeted conservation strategies in Southeast Asian countries to preserve these vital corridors in the face of increasing environmental threats.

Given the escalating threats facing *N. nebulosa* under both current and projected future conditions, there is an urgent need to implement a comprehensive, proactive, and landscape-level management plan to ensure the long-term conservation of this species and other colligated felids inhabiting its range [33,101]. In response, the present study advocates for the establishment of a detailed protection action plan at the national level, with an emphasis on strengthening transboundary cooperation. Such a collaborative approach is essential to effectively safeguarding this species across its range, particularly in regions where habitat and corridors span multiple countries, demanding coordinated conservation efforts between neighboring nations. A key aspect of this strategy involves expanding the network of PAs within the species’ distribution. Moreover, various transboundary initiatives have already been implemented across this region for different wildlife species. These initiatives encompass a range of coordinated actions, including joint patrols, increased community involvement, anti-poaching operations, intelligence-sharing mechanisms, and concerted efforts to maintain forest connectivity and reduce habitat fragmentation. Such integrated approaches have led to significant progress in reducing illegal activities such as logging and poaching while also facilitating the restoration of essential habitats and ecological corridors, which have, in turn, contributed to a notable recovery in wildlife populations. Some of the notable transboundary efforts in this specific region include the India–Nepal Terai Arc Landscape (TAL), the India–Bhutan Manas Tiger Reserve, and the Thailand–Myanmar Tenasserim Range. Hence, it is critical that potential habitats within regions classified as extinct or possibly extinct are ground-truthed through field validation to assess their suitability as future refuges for the species. This approach would not only help identify new conservation opportunities but also ensure that areas with high potential for recolonization or reintroduction are preserved. Furthermore, this study also highlights the alarming loss of corridor connectivity, which poses a significant threat to the species’ genetic diversity. Moreover, recent research has highlighted that reduced genetic diversity, combined with the accumulation of harmful genetic mutations, is strongly associated with a decline in reproductive success [102]. Therefore, protecting and maintaining the biological corridors identified in this study is crucial for mitigating genetic isolation and ensuring the species’ long-term viability. The preservation of these corridors would allow for the natural movement of individuals, which is essential for gene flow and the maintenance of genetically healthy populations. Furthermore, it is imperative to conduct ground-level assessments based on the results of the corridor connectivity between protected areas (PAs) and suitable patches to gain a clearer understanding of the movement pathways utilized by this species. The connectivity of these corridors may be disrupted by various anthropogenic factors such as urban development, agricultural activities, and the construction of roads, all of which require thorough evaluation to formulate site-specific conservation measures. Moreover, ensuring the protection of these critical movement corridors and initiating restoration projects where necessary is vital to maintaining habitat connectivity. Additionally, expanding and reinforcing these corridors by creating buffer zones can enhance the species’ mobility, especially in the event of habitat interruptions, and improve the corridors’ resilience to climate change. Such measures must consider climatic, geographical, and ecological aspects to ensure long-term effectiveness. A transboundary corridor management plan is also essential to maintain ecological continuity across international borders, allowing the species to migrate and adapt to shifting prey availability and changing climate conditions. Hence, the lessons learned from the successful implementation of similar strategies for other species (Yellowstone to Yukon Conservation Initiative in North America and Canada, the Tiger Corridor Project in India, and the Jaguar Corridor Initiative in South America) can provide valuable insights and inform conservation efforts for this species as well. In addition to the protection of corridors, safeguarding large, continuous forested areas is critical. The increasing prevalence of commercial logging in these regions is a serious concern, as it contributes to the fragmentation of critical habitats. Moreover, forest loss may drive the species out of its natural habitat, exposing it to higher risks of poaching or human–wildlife conflict. Therefore, efforts must be made to protect these forested areas from further degradation. Moreover, phylogeographic studies across various regions are crucial to assessing the genetic diversity of the species, ensuring the identification of distinct populations that can be strategically selected for breeding programs. Such assessments are essential for reintroduction efforts, as they will enhance genetic diversity and viability, thereby expanding the gene pool and improving the long-term resilience of the population. This genetic insight will aid in maintaining healthy, genetically diverse populations, which is vital for the species’ adaptive potential and overall survival, especially in the context of habitat fragmentation and changing environmental conditions. Alongside these conservation efforts, it is equally crucial to actively involve local communities residing near *N. nebulosa* habitats through awareness campaigns aimed at reducing hunting and the illegal trade of this species. These programs should not only highlight the importance of biodiversity conservation but also promote alternative livelihoods, addressing the underlying socio-economic factors that drive wildlife exploitation. The illegal trade of *N. nebulosa* body parts continues to be a major threat, with numerous records of confiscations. This underscores the need for increased vigilance by law enforcement agencies, particularly in monitoring and disrupting trade routes known to facilitate wildlife trafficking. Strengthened enforcement of wildlife protection laws, along with cross-border cooperation, is vital for combating illegal trade. Further, designating *N. nebulosa* as a flagship species could further enhance its conservation efforts by raising its profile and rallying public and governmental support. As a flagship species, *N. nebulosa* would not only benefit from increased attention and resources but also serve as an umbrella species, helping to protect other sympatric felids that share its habitat. This approach has been found effective in other cases where the designation of a flagship species has facilitated the conservation of entire ecosystems. Accordingly, there is an urgent need to identify a flagship species for evergreen forests, which could be served by designating the *N. nebulosa* as the flagship, which would lead to conservation efforts that also benefit sympatric species within the same habitat, fostering broader ecosystem protection. Therefore, prioritizing and implementing these measures could significantly enhance the conservation of this species within its range and help ensure its survival in the evergreen forests of South and Southeast Asia.

## 5. Conclusions

This study presents a thorough evaluation of the threats confronting *N. nebulosa* across its existing and historical ranges while also identifying potential suitable habitats within these areas. The research highlights significant challenges to the species’ survival, including habitat loss, fragmentation, and reduced corridor connectivity, which are increasingly intensified by the effects of climate change. These issues are especially alarming in light of the escalating threats posed by biodiversity loss across the region, which not only jeopardize the survival of the clouded leopard but also imperil other sympatric felid species. Therefore, the findings underscore the urgent need for a coordinated and multifaceted conservation strategy to ensure the long-term protection of *N. nebulosa*. Furthermore, this study emphasizes the necessity of implementing practical measures to address the species’ shrinking range, particularly considering its declining genetic diversity and increased vulnerability to inbreeding depression. Moreover, protecting viable forest patches alongside the corridors that maintain connectivity between them is critical to mitigating these threats. The findings and recommendations of this research offer essential resources for scientists and conservationists to implement targeted conservation strategies in the suitable areas delineated by this study, considering field validation across South and Southeast Asia. Moreover, this research establishes a robust foundation for developing a comprehensive conservation and management plan tailored to the unique needs of *N. nebulosa* populations throughout the region.

## Figures and Tables

**Table 1:** Model fit metrics for each of the participating modeling methods and for the final ensemble model for estimation of habitat suitability of clouded leopard. A total of five model algorithms, i.e., Maximum Entropy (MaxEnt), Boosted Regression Tree (BRT), Generalized Linear Model (GLM), Multivariate Adaptive Regression Splines (MARS), and Random Forest (RF). AUC: Area under Curve, ΔAUC: Change in Area under Curve (Training Cross Validation), PCC: Proportion Correctly Classified, TSS: True Skill Statistic.

Model	Dataset	AUC	ΔAUC	PCC	TSS	Kappa	Specificity	Sensitivity
BRT	Train	0.972	0.125	92.9	0.856	0.853	0.932	0.924
	CV	0.847		75.7	0.51	0.505	0.777	0.733
GLM	Train	0.925	0.093	84	0.679	0.672	0.842	0.837
	CV	0.832		74.2	0.477	0.474	0.775	0.702
MARS	Train	0.909	0.105	83.6	0.672	0.664	0.835	0.837
	CV	0.804		72.8	0.465	0.452	0.73	0.735
MaxEnt	Train	0.95	0.109	88.8	0.778	0.771	0.886	0.891
	CV	0.841		78.4	0.565	0.558	0.809	0.755
RF	Train	0.866	0.016	80	0.598	0.591	0.805	0.793
	CV	0.882		77	0.531	0.526	0.797	0.733

**Table 2:** The mean percentage contribution of the covariates generated from the final ensemble model for the clouded leopard.

Predictor	Abbreviations	BRT	GLM	MARS	MAXENT	RF	Mean	Mean %
Precipitation Seasonality (Coefficient of Variation)	bio_15	0.030	0.000	0.116	0.029	0.000	0.035	8.43
Precipitation of Wettest Quarter	bio_16	0.021	0.000	0.065	0.010	0.023	0.024	5.75
Precipitation of Warmest Quarter	bio_18	0.000	0.110	0.037	0.005	0.000	0.030	7.33
Precipitation of Coldest Quarter	bio_19	0.024	0.000	0.000	0.037	0.007	0.014	3.28
Mean Diurnal Range (Mean of monthly (max temp—min temp))	bio_2	0.029	0.113	0.023	0.058	0.000	0.045	10.70
Temperature Annual Range	bio_7	0.145	0.267	0.085	0.258	0.023	0.155	37.37
Built-up	builtup	0.000	0.040	0.000	0.019	0.000	0.012	2.87
Cropland	cropland	0.037	0.067	0.044	0.023	0.000	0.034	8.19
Elevation	elevation	0.000	0.000	0.000	0.039	0.000	0.008	1.87
Evergreen Forests	evergreen_for	0.000	0.092	0.000	0.070	0.040	0.041	9.74
Mixed/Deciduous Forest	mixed_for	0.000	0.013	0.000	0.021	0.010	0.009	2.10
Slope	slope	0.008	0.000	0.000	0.040	0.001	0.010	2.35

**Table 3:** This table represents the suitable habitat extent (in sq. km) for the clouded leopard within its IUCN range under present and future climatic scenarios.

Scenario	Overall Range	Extant	Possibly Extant	Extinct	Possibly Extinct	Protected Areas
Present	93,353	44,033	20,034	14,022	15,264	25,614
SSP 245 (2041–2060)	68,171	37,706	7349	11,008	12,110	24,327
SSP 245 (2061–2080)	61,146	33,804	6107	10,198	11,038	22,352
SSP 585 (2041–2060)	61,666	36,200	4599	10,245	10,623	23,576
SSP 585 (2061–2080)	54,968	31,830	3841	9732	9565	21,217

**Table 4:** The mean habitat suitability for clouded leopard in both current and future scenarios within different countries (descending order) in the entire IUCN extant. The gain is represented by “+”, whereas the loss is represented by “−”. GR: growth rate.

Country	Present	SSP 245 (2041–2060)	GR of SSP 245 (2041–2060) from Present	SSP 245 (2061–2080)	GR of SSP 245 (2061–2080) from Present	SSP 585 (2041–2060)	GR of SSP 585 (2041–2060) from Present	SSP 585 (2061–2080)	GR of SSP 585 (2061–2080) from Present
Malaysia	+0.743	+0.672	−9.54	+0.662	−10.88	+0.659	−11.25	+0.649	−12.59
Laos	+0.493	+0.352	−28.62	+0.342	−30.68	+0.323	−34.47	+0.315	−36.10
India	+0.488	+0.261	−46.43	+0.251	−48.53	+0.234	−52.02	+0.214	−56.12
Cambodia	+0.410	+0.330	−19.44	+0.321	−21.70	+0.291	−29.03	+0.276	−32.69
Nepal	+0.404	+0.340	−15.83	+0.330	−18.31	+0.311	−23.07	+0.299	−26.04
Vietnam	+0.365	+0.279	−23.44	+0.268	−26.46	+0.259	−29.05	+0.243	−33.43
Myanmar	+0.343	+0.232	−32.42	+0.222	−35.35	+0.201	−41.33	+0.199	−41.91
Thailand	+0.317	+0.259	−18.51	+0.249	−21.66	+0.241	−24.05	+0.226	−28.78
Bhutan	+0.273	+0.232	−14.93	+0.222	−18.59	+0.210	−23.14	+0.189	−30.83
Bangladesh	+0.242	+0.215	−11.35	+0.205	−15.48	+0.195	−19.46	+0.188	−22.35
China	+0.098	+0.093	−5.39	+0.092	−6.42	+0.089	−9.15	+0.083	−15.27

**Table 5:** Assessment of habitat shape geometry of clouded leopard in present and future scenarios. NP: no. of patches, PD: patch density, LPI: largest patch index, TE: total edge, LSI: landscape shape index, AI: aggregation index.

Scenario	NP	PD	LPI	TE	LSI	AI
Present	468	993,511	2.767	640.794	26.4291	82.1631
SSP 245 (2041–2060)	429	913,023	2.420	525.378	24.5273	70.399
SSP 245 (2061–2080)	385	819,379	2.200	477.288	23.4691	66.2725
SSP 585 (2041–2060)	378	804,481	2.349	506.31	23.8346	62.7976

**Table 6:** Assessment of corridor connectivity of clouded leopard within the entire IUCN range in both the current and future climate change scenarios. The gain is represented by “+”, whereas the loss is represented by “−”. CAM: Cambodia; LAO: Laos; BHU: Bhutan; IND: India; MYA: Myanmar; BAN: Bangladesh; CHI: China; VIET: Vietnam; THA: Thailand; MAL: Malaysia; GR: Growth Rate; SSP: Shared Socioeconomic Pathways.

Corridors	Present	SSP 245 (2041–2060)	GR of SSP 245 (2041–2060) from Present	SSP 245 (2061–2080)	GR of SSP 245 (2061–2080) from Present	SSP 585 (2041–2060)	GR of SSP 585 (2041–2060) from Present	SSP 585 (2061–2080)	GR of SSP 585 (2061–2080) from Present
BHU_IND	+2.441	+2.063	−15.48	+1.901	−22.10	+1.802	−26.16	+1.662	−31.89
NEP_IND	+2.114	+1.901	−10.09	+1.719	−18.67	+1.600	−24.33	+1.490	−29.53
MYA_CHI	+1.140	+0.996	−12.60	+0.886	−22.27	+0.825	−27.62	+0.801	−29.73
NEP_CHI	+1.044	+0.767	−26.48	+0.657	−37.02	+0.644	−38.30	+0.621	−40.51
IND_BAN	+1.041	+0.909	−12.76	+0.709	−31.96	+0.689	−33.88	+0.656	−37.01
CHI_LAO	+0.938	+0.797	−15.01	+0.697	−25.67	+0.657	−29.97	+0.622	−33.70
MYA_LAO	+0.881	+0.548	−37.88	+0.491	−44.35	+0.455	−48.37	+0.423	−52.00
MYA_THA	+0.754	+0.593	−21.32	+0.489	−35.16	+0.421	−44.18	+0.409	−45.77
BHU_CHI	+0.614	+0.375	−39.00	+0.289	−52.93	+0.269	−56.19	+0.235	−61.73
LAO_VIET	+0.612	+0.420	−31.43	+0.400	−34.64	+0.370	−39.54	+0.343	−43.95
BAN_MYA	+0.545	+0.397	−27.16	+0.376	−31.05	+0.343	−37.10	+0.321	−41.14
LAO_CAM	+0.446	+0.356	−20.15	+0.333	−25.31	+0.314	−29.57	+0.301	−32.49
CHI_VIET	+0.347	+0.275	−20.77	+0.234	−32.51	+0.212	−38.86	+0.198	−42.90
THA_LAO	+0.343	+0.231	−32.59	+0.211	−38.53	+0.199	−42.02	+0.177	−48.43
IND_CHI	+0.339	+0.233	−31.25	+0.209	−38.26	+0.189	−44.17	+0.156	−53.92
LAO_VIET	+0.302	+0.209	−30.82	+0.190	−37.10	+0.170	−43.72	+0.140	−53.65
THA_CAM	+0.266	+0.170	−36.04	+0.160	−39.76	+0.140	−47.29	+0.120	−54.82
THA_MAL	+0.253	+0.176	−30.31	+0.157	−37.89	+0.136	−46.20	+0.122	−51.74

## Data Availability

Data used for the analysis were sourced from open-access resources.

## Supplementary Materials

The following supporting information can be downloaded at: https://www.mdpi.com/article/10.3390/biology13110902/s1, Figure S1. Figure showing the correlation between the covariates chosen for the final model for clouded leopard; Figure S2. Evaluation Matrix performance across model runs for clouded leopard. Brown—represents the correlation coefficient among the five different models. Yellow—represents the proportion of deviance explained; Green—represents the proportion of correctly classified; Blue—represents the area under curve (AUC); and Pink—represents true skill statistics; Figure S3. Confusion matrixes, model calibration plots, and residual plots for clouded leopard. Row 1 represents a spatial pattern of residuals, where the color ramp indicates the magnitude of deviance and size represents the quantity. Row 2 represents the model calibration plot across all five different models for cross-validation split. Row 3 represents the confusion matrix for all five models, plotted by observed vs. predicted, where the color ramp from the lowest value of 0% (white) to 100% (red) indicates the quantification of particular pair types. Column a. represents plots for BRT, Column b. represents plots for GLM, Column c. represents plots for MARS, and Column d. represents plots for MaxEnt; Figure S4. The response curves of the covariates selected by each of the participating ensemble model for clouded leopard. (A) BRT, (B) GLM, (C) MARS, (D) MaxEnt, and (E) RF.
