# Supplementary material for: Forecasting Suitable Habitats of the Clouded Leopard (Neofelis nebulosa) in Asia: Insights into the Present and Future Climate Projections Within and Beyond Extant Boundaries
Source: Biology (Basel). 2024 Nov 5;13(11):902. doi: 10.3390/biology13110902 (PMC11592018; doi:10.3390/biology13110902)
Supplement: Supplementary file 1 [file biology-13-00902-s001.zip › biology-3264410-supplementary.pdf]

# Supplementary Materials

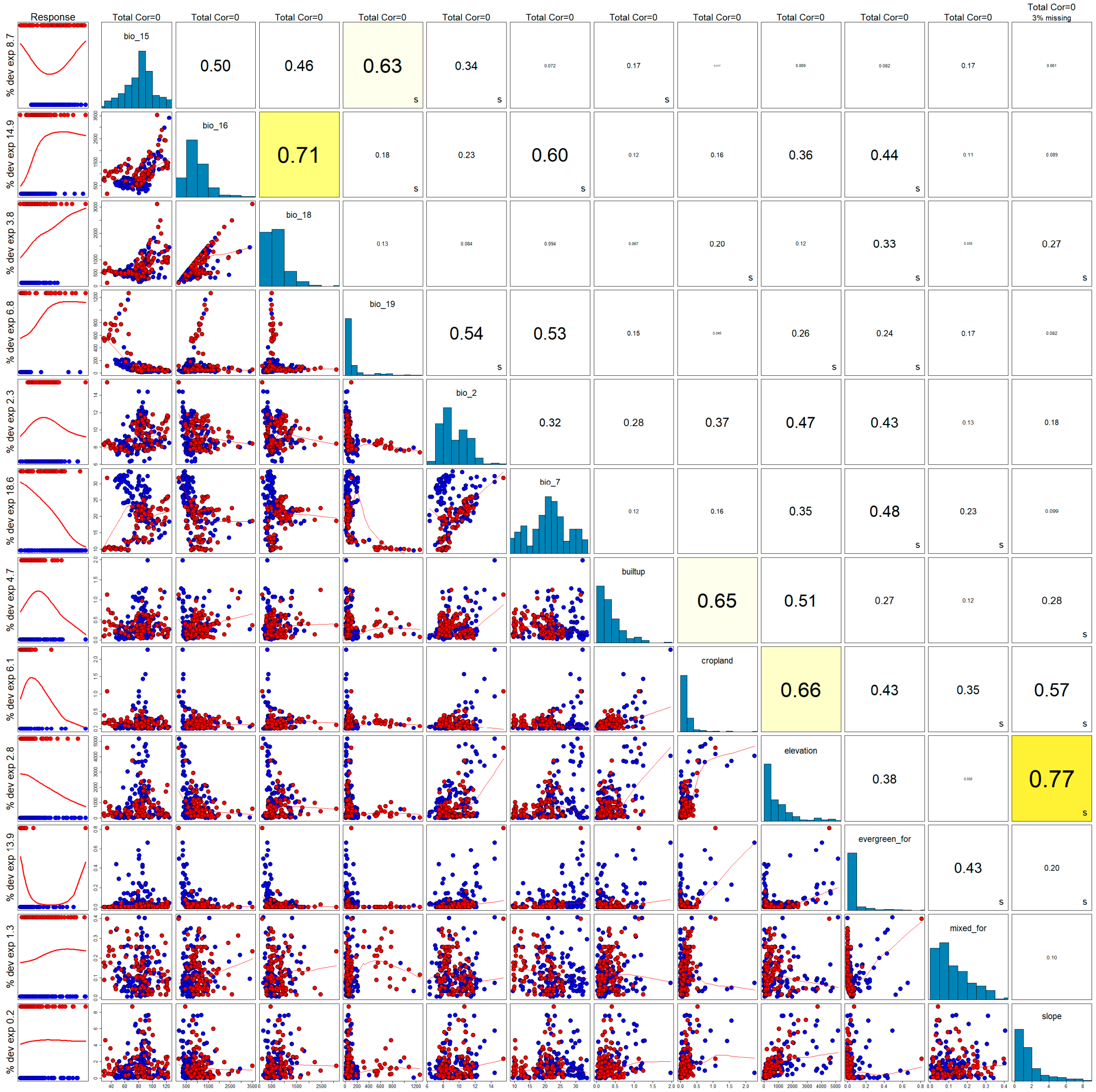


**Figure S1.** Figure showing the correlation between the covariates chosen for the final model for clouded leopard.

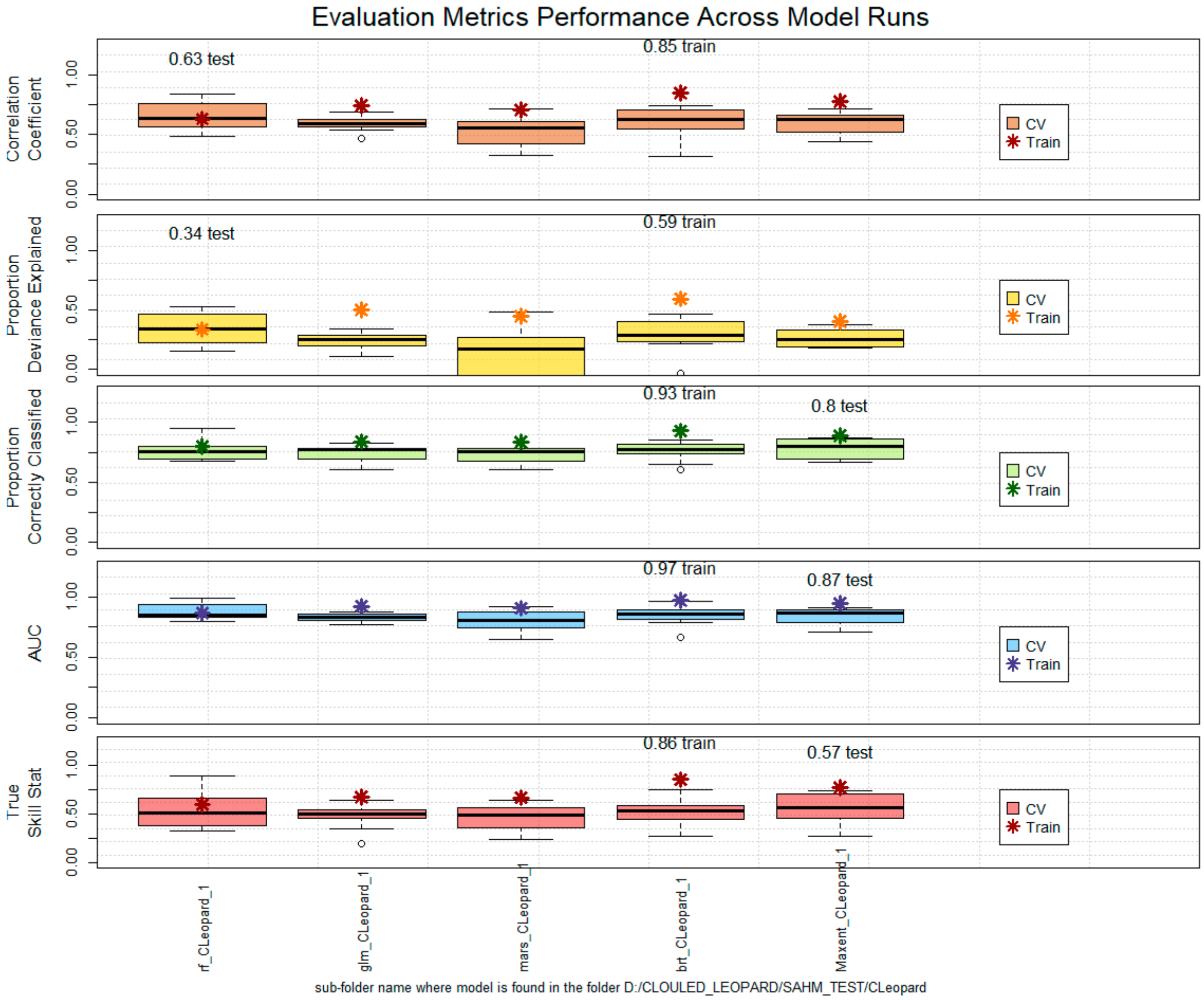


**Figure S2.** Evaluation Matrix performance across model runs for clouded leopard. Brown - represents the correlation coefficient among the five different models. Yellow - represents the proportion of deviance explained; Green - represents the proportion of correctly classified; Blue - represents the area under curve (AUC); and Pink - represents true skill statistics.

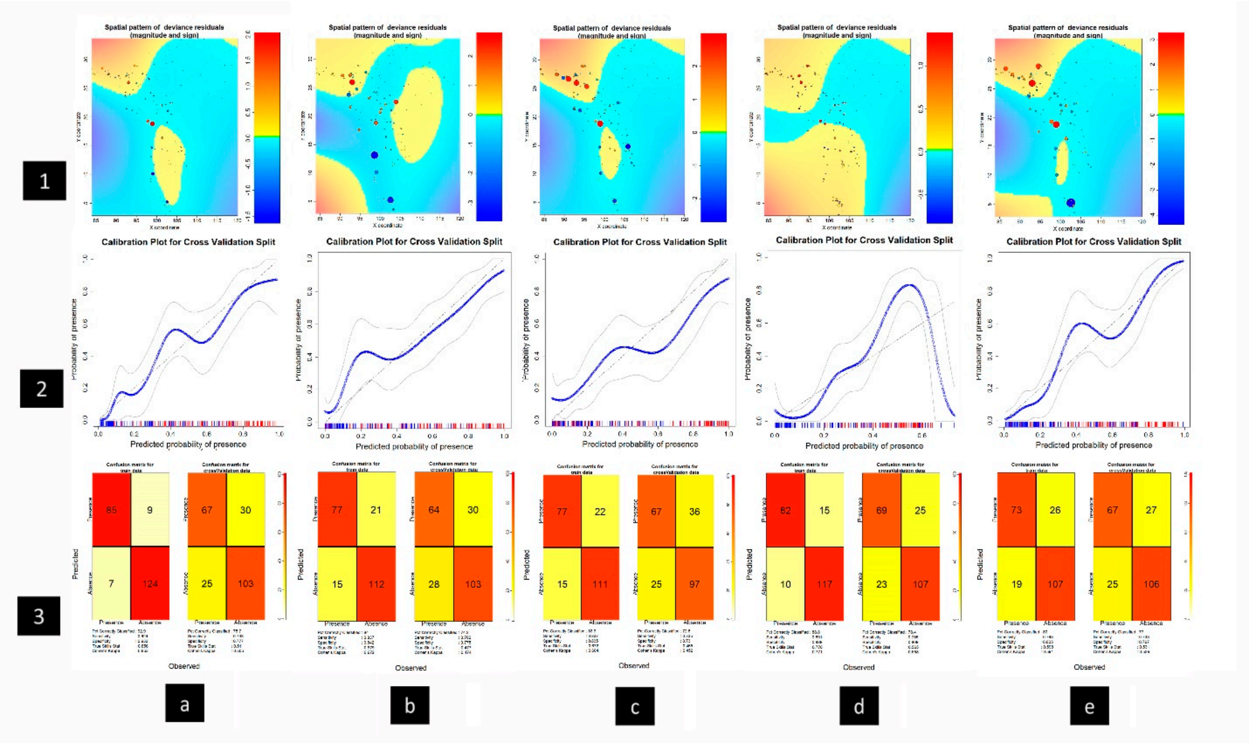


**Figure S3.** Confusion matrixes, model calibration plots and residual plots for clouded leopard. Row 1 represents a spatial pattern of residuals where the color ramp indicates the magnitude of deviance and size represents the quantity. Row 2 represents the model calibration plot across all five different models for cross-validation split. Row 3 represents the confusion matrix for all five models, plotted by observed vs. predicted, where the color ramp from the lowest value of 0% (white) to 100% (red) indicates the quantification of particular pair types. Column a. represents plots for BRT, Column b. represents plots for GLM, Column c. represents plots for MARS, and Column d. represents plots for MaxEnt.

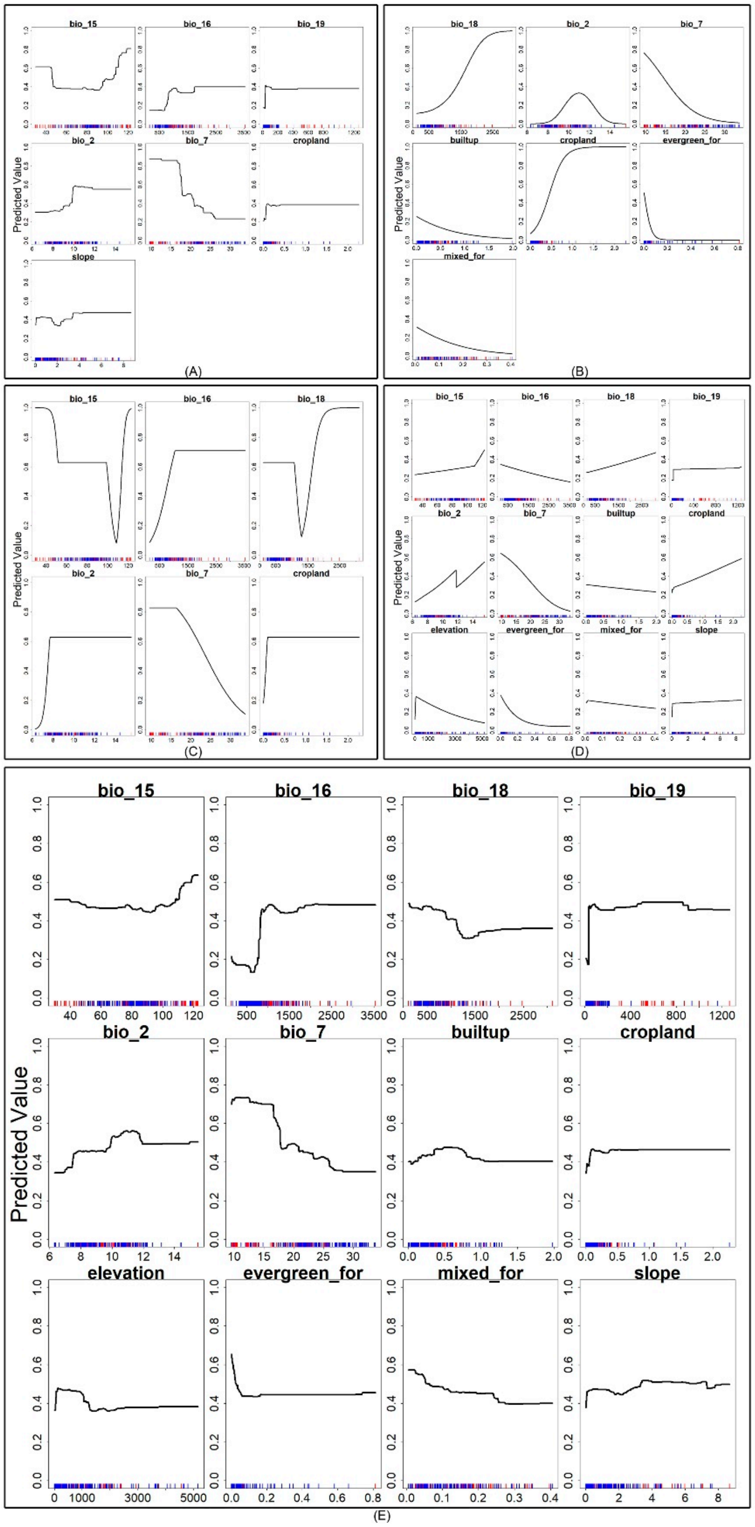


**Figure S4.** The response curves of the covariates selected by each of the participating ensemble model for clouded leopard. (A) BRT, (B) GLM, (C) MARS, (D) MaxEnt, and (E) RF.
